# A Survey of Greek Primary Care Physicians on Their Likeability in Treating Migraines and Other Common Diseases

**DOI:** 10.3390/medicina59040734

**Published:** 2023-04-09

**Authors:** Michail Vikelis, Andreas A. Argyriou, Anastasia Antoniou, Konstantinos C. Spingos, Athanasios E. Skliros, Konstantinos Bilias, Aikaterini Kouroudi, Emmanouil V. Dermitzakis, Efstathios A. Skliros

**Affiliations:** 1 Headache Clinic, Mediterraneo Hospital, 16673 Glyfada, Greece; 2 Neurology Department, Agios Andreas State General Hospital of Patras, 26332 Patras, Greece; andargyriou@yahoo.gr; 3 2nd Department of Psychiatry, “Attikon” University General Hospital, School of Medicine, National and Kapodistrian University of Athens, 12462 Athens, Greece; 4 Corfu Headache Clinic, 49131 Corfu, Greece; 5 Hellenic Society for Primary Care Research and Continuing Education, 11528 Athens, Greecestathis.skliros@gmail.com (E.A.S.); 6 Greek Society of Migraine and Headache Patients, 11251 Athens, Greece; 7 Euromedica General Clinic, 54645 Thessaloniki, Greece; manolis.dermitzakis@gmail.com; 8 Nemea Health Center, 20500 Corinthia, Greece

**Keywords:** primary care physicians, migraine, neurological disorders, likeability

## Abstract

*Background and objectives*: Migraine is considered the most clinically important primary headache due to its high prevalence and significant burden. Although globally categorized as one of the leading causes of disability, it is still largely underdiagnosed and undertreated. Worldwide, migraine care is in most cases provided by primary care physicians. The aim of our study was to assess the attitudes of Greek primary care physicians toward treating migraine compared to other common neurological and general medical disorders. *Methods*: We surveyed 182 primary care physicians with the use of a 5-point questionnaire regarding their preference in treating ten common medical conditions, including migraine, hypertension, hyperlipidemia, upper respiratory tract infections, diabetes mellitus, lower back pain, dizziness, transient ischemic attack, diabetic peripheral neuropathy, and fibromyalgia. *Results*: Overall, with regards to preference to treat, migraine scored very low (3.6 ± 1.0), next to diabetic peripheral neuropathy (3.6 ± 1.0), and third from the bottom to fibromyalgia (3.25 ± 1.06). In contrast, physicians reported a much higher preference to treat hypertension (4.66 ± 0.60) and hyperlipidemia (4.6 ± 1.0). *Conclusions*: Our results indicate that Greek primary care physicians dislike treating migraines but also other neurological diseases. Topics for further investigation include the reasons for this dislike, any associations with poor patient satisfaction, treatment results, or both.

## 1. Introduction

Primary headaches, including migraines and tension-type headaches, are among the most common neurological disorders in everyday clinical practice. It is estimated that migraine affects up to 12% of the general population in western countries and rises to almost 18% in women of reproductive age [1,2]. It is also ranked among the leading causes of disability, as measured by years lived with disability (YLD), according to the Global Burden of Disease Study 2019 [3,4].

Migraine diagnosis is based on clinical criteria and is generally considered an easy task for a skilled and experienced physician. However, in some cases or if the physician is not trained properly or lacks experience, the diagnosis may be challenging, as migraine is characterized by great clinical heterogeneity. Pain may be unilateral or bilateral and may be located in every part of the head, including the face or the occipital area. In some patients, location is fixed, while in others it may change from attack to attack. While throbbing pain is quite typical and common, many patients never experience migraine pain like this, and they report their pain as being of a pressing quality. The typical duration of untreated attacks is 4 to 72 h, but longer attacks may also occur in some patients. The hallmark of migraine features are the clinical characteristics of photophobia and phonophobia. Both may vary significantly in intensity from patient to patient. Some may experience minimal symptoms, while others cannot tolerate any sound or light. Nausea is commonly present but also varies in intensity, while vomiting is less common but still regarded as a core migraine feature. Moreover, the clinical characteristics of an individuals’ migraine may change significantly through the years. All this heterogeneity may be confusing if the physician has the stereotypical “textbook” presentation of unilateral headache, throbbing pain, severe sensitivity to light and sound, and nausea and vomiting in mind as the clinical picture of migraine attacks [3]. Migraine can be episodic (less than 15 days monthly) or chronic (more than 15 headache days monthly, of which at least eight are of the migrainous type or respond to migraine-specific medication, for more than 3 months), with the disease’s phenotype in patients with chronic migraine (CM) generally considered less benign compared to the episodic type of migraine, as CM is characterized by a longer average duration of headache, more pain intensity, moderate/severe pain-associated autonomic symptoms, and increased pain-related comorbidities than episodic migraine [4]. Tension-type headache (ΤΤH), estimated to affect around 1.6 billion people worldwide, is characterized by evidence of a dull, steady ache bilateral headache that is not accompanied by nausea, vomiting, or sensitivity to noise and light. Generally, the TTH-associated disability is much less severe compared to migraine [3].

Worldwide, primary care physicians are commonly the first point of contact for patients and families with the health care system, providing consultation for the management of health diseases and controlling the referral of patients to specialists when needed. They are also the central hub for disease prevention and the promotion of healthy living. Primary care physicians practice general/family medicine and are trained in the certified medical specialty of general medicine to deal with any health problem, including neurological diseases, at private practice offices, primary health care facilities, the hospital, or the patient’s home [5].

While in most countries general/family medicine is practiced by general practitioners, in Greece board-certified internists may also work as primary care physicians and, as a matter of fact, have actually been the majority of primary care physicians until recently. Primary care physicians may work at public or private primary health care facilities. A competent primary care physician is warranted to likely buffer to some extent the negative effects of both the economic crisis and the COVID-19 pandemic that recently beset Europe and Greece on vulnerable people’s health, particularly their mental health [6,7]. An early recognition and provision of sufficient treatment of the most common and relatively easily identifiable neurological diseases, such as primary headaches, that can be significantly affected by the individual’s mental health status should also be among the objectives of primary care medicine.

According to the available evidence, there is a lack of key characteristics for a strong primary care organization in the current Greek healthcare system, and as such, there is a need for major reforms to strengthen it [8]. Among other actions, this could be accomplished by providing a more comprehensive education curriculum to general practitioners during their residency training in various fields, especially in diseases affecting the nervous system. Currently, a five-year residency program in general medicine applies in Greece, during which residents are trained for just one month in neurology; prior to this, there was a four-year program with no neurology training at all [9]. As such, a training remodel is warranted in order to provide basic knowledge to general practitioners on common mental and neurological diseases [10]. The internal medicine residency program in Greece includes training and hands-on experience in certain neurological conditions, such as stroke, but not in other common neurological conditions that are mainly treated on an outpatient basis, such as migraine. Given that general practitioners in training spend a significant amount of time in internal medicine departments, their experience with neurological conditions comes mainly, if not completely, from this period of their training and refers to neurological conditions that may be encountered in an internal medicine ward.

Although primary headaches, including migraine, are among the most common and potentially disabling disorders encountered in clinical practice [1,2,3], there is evidence to suggest that many family doctors remain reluctant or even dislike treating these patients. In support of the latter point of view are the results of a survey of 148 family doctors that aimed to assess whether family doctors in the US like to treat migraine and other common disorders. The participants reported that they like to treat general medical conditions much more than migraine and other neurological diseases [11]. Noteworthy, these interesting findings have not yet been reproduced internationally.

As such, whether family doctors like to treat patients with migraines remains vaguely explored, despite the fact that in many countries worldwide, primary care doctors significantly contribute to the care of those suffering with migraines [12,13]. Lack of sufficient training and experience could rank among the possible factors significantly influencing the attitudes of general practitioners towards migraine. Concerns about diagnostic pitfalls and inadequate response to a given symptomatic or prophylactic treatment leading to reduced patients’ satisfaction, abandoning of follow-up schedules, and relying on over-the-counter medications to provide adequate pain relief and reduce disability [14] could be an explanation for this.

In Greece, patients with any medical condition may consult primary care physicians or self-refer to specialists (neurologists and headache specialists), both in the public and private healthcare sectors. However, the management of migraine is considered to be mainly provided by primary care physicians, as indicated by the relatively high number of Greek migraine patients, which is estimated at about one million [15], compared to the low number of neurologists (around 1000 throughout the country). In addition, neurologists are mostly located in urban centers and headache specialists in large cities. Notably, there are semi-urban or rural areas, including the numerous Greek islands, where accessing specialists remains difficult. Hence, surveying whether the general practitioners’ or internists’ dislike or show indifference to migraine in Greece, as much as in the US [11], is not only intriguing but it might add a crucial component of insight. It may be because the preference to treat migraine may depend on both cultural and intrinsic factors, i.e., difficulties in establishing an accurate migraine diagnosis or complexities in finding efficient therapeutic options in difficult-to-treat migraineurs. The objective of the current survey was to ascertain the latter open issue by evaluating the preference of Greek primary care physicians to treat common medical conditions, with an emphasis on migraine.

## 2. Materials and Methods

Our target study population consisted of board-certified primary care physicians, either general practitioners or internal medicine specialists, currently attending the Continuing Medical Education program of the Hellenic Society of Primary Care Research and Continuing Education. Participants attended 3 days of seminars covering various topics, including a one-hour lecture on migraine, during which basic migraine aspects were reviewed, including epidemiology, a summary of pathophysiology, the International Classification of Headache Disorders, 3rd edition (ICHD-III) diagnostic criteria [16], and options for symptomatic and prophylactic treatment.

From a total of 355 attending primary care physicians, 182 agreed to participate and complete the survey questionnaire, corresponding to a response rate of 51.3%. The survey questionnaire consisted of a total of 17 items, including demographics (2 items), work status (4 items), participants’ personal history of migraine or tension-type headache (1 item) according to the ICHD-III criteria, as well as questions concerning their likeability to treat migraine and nine other diseases and symptoms that are encountered in primary care settings. When mentioning “a personal history of migraine or tension-type headache”, we are referring to participants having been personally (not their family members) diagnosed with the latter primary headache disorders.

Participating primary care physicians were asked to respond to the statement “I like to treat patients with this disease or symptom” with the use of a five-point Likert scale (1: strongly disagree; 2: disagree; 3: neutral; 4: agree; 5: strongly agree). This methodology is considered generally appropriate for a survey [8], while for comparison purposes we used the same set of disorders and symptoms as those used in the only previously published survey by Evans et al. [11].

All study procedures were in accordance with the 1964 Helsinki Declaration and its amendments, the EU General Data Protection Regulation (679/2016). Ethics approval and written informed consent are not required in questionnaire-based investigations. The provision of written information about the study, along with the questionnaire and voluntary participation, provided implied consent. Participating physicians completed the questionnaires fully anonymously.

### Statistics

Descriptive statistics were computed for all variables and, depending on their nature, generated categorical variables (observed counts and weighted percentages) and continuous variables (mean or median with the corresponding standard error or range). One-way analysis of variance (ANOVA) and student *t*-tests were used for intra- and inter-group comparisons of continuous variables. Pearson’s chi-squares test was used to compare differences in proportions between groups. A *p*-value < 0.05 was deemed statistically significant. SPSS for Windows (release 27.0; SPSS Inc., Chicago, IL, USA) was utilized to perform the statistical analyses.

## 3. Results

The mean age ± SD of our 182 surveyed participants was 49.6 ± 9.1 years (range 30–70 years). There were 110 males (60.4%). Surveyed physicians were almost equally distributed between those working in the private sector and those who were employees of the Greek National Health System. Among participants, 95 (52.2%) were general practitioners and 87 (47.8%) were internists working in primary care. The demographic characteristics and work status of participants are summarized in Table 1.

A personal history of migraine and TTH was reported by 8.25% (n = 15) and 10.45% (n = 19) of the participants, respectively, with no gender predominance between them (*p* = 0.137). Amongst participants who had been personally diagnosed with TTH vs. those with a personal history of migraine diagnosis, there were no differences in age (46.9 ± 8.1 vs. 47.0 ± 9.1; *p* = 0.98); in years of practicing medicine (9.8 ± 7.1 vs. 11.1 ± 8.7; *p* = 0.659); and in working status, i.e., private vs. public sector (x^2^ = 0.098; *p* = 0.755). The prevalence of migraine and TTH history among survey respondents, categorized by gender, is presented in Table 2.

Table 3 shows the responses (means and SD on the 5-point Likert scale) to the statement “I like to treat patients with this disease or symptom” for each of the 10 diseases or symptoms. Overall, migraine scored very low (3.6 ± 1.0), equally to diabetic peripheral neuropathy. Only fibromyalgia scored lower (3.2 ± 1.0), while, interestingly, transient ischemic attacks were liked more (3.9 ± 1.0). Our results showed that participating physicians liked to treat patients with general medical conditions, such as hyperlipidemia, hypertension, diabetes, and upper respiratory infections, significantly more than patients with neurological conditions.

Of note, respondents with a personal history of either lifetime migraine with/without aura or TTH reported a comparable likeability to treat migraine compared to those without a history of headache (*t* = 1.12; *p* = 0.12), correcting for unequal variance. There were no differences between general practitioners and internists working in primary care.

## 4. Discussion

Physicians’ likeability to a medical condition refers to their high subjective positive attitudes and beliefs to treat the disorder. In the current survey, we assessed the likeability of physicians working in primary care settings in Greece to treat migraine and other common disorders or symptoms. It has been previously suggested that a dislike for migraine might be a factor that could negatively influence patients’ satisfaction and result in the withholding of treatment [14]. The response rate of our survey (51.3%) was comparable to other surveys with similar study designs [11,17], thoroughly supporting the validity and generalizability of results overall about the likeability of Greek primary care doctors in treating certain diseases. Despite this, further research on methodology for physician surveys is warranted in order to increase the response rates and diminish non-response bias.

Our main finding is that primary care physicians liked to treat general medical conditions more than neurological ones, including migraines. With the exception of fibromyalgia (3.2 ± 1.0) being the least liked, migraines were the second least liked condition to treat (3.6 ± 1.0) than any other medical or neurological condition or symptom included in the survey. The likeability of treating diabetic peripheral neuropathy (3.6 ± 1.0) was identical to migraine. Our findings are generally in accordance with the findings of the survey of US primary care physicians, which demonstrated a low likeability rating for migraine and other neurological diseases compared to other more general conditions, including hypertension and hyperlipidemia [11]. Given that migraine is a highly prevalent disorder [1,2,3,4] and that a significant part of care for migraine patients is provided by primary care physicians [12,13], the fact that treating migraines is not popular may herald a significant issue, as the management may be suboptimal and result in low patient satisfaction rates.

Although specific answers about the possible effects of the physicians’ preferences on their medical performance are lacking in Greece, one could claim that several factors may account for the low likeability of primary care doctors to treat neurological diseases, including migraine. The lack of training in the topic and the time-consuming nature of visits by migraine patients may be two examples. In addition, the geographical distribution of the islandic or rural Greek population could result in a lack of specialized neurologists in isolated residential areas or even in towns as well as in small cities. This results in a situation where a significant proportion of those suffering with migraines may not have access to a neurologist or a headache clinic and be solely dependent on primary care physicians for migraine pain relief and prophylaxis. This may lead to a performance bias against primary care physicians as, in difficult or non-responding cases, referring patients to secondary care for help is not easy.

We were unable to demonstrate a positive correlation between our surveyed doctors’ personal medical history of migraine and their preference to treat this condition. As such, a personal history of migraine was not associated with increased likeability to treat it, similarly to the results of a survey in neurologists that showed that neurologists with or without a personal history of migraine liked to treat migraines comparably [18]. Considering our sample size, which might not have allowed positive results to be extrapolated, the latter is an important finding because it suggests that doctors’ personal experience with a disease may not necessarily improve patients’ care or satisfaction, resulting from an increased sense of reassurance when informed that their treating physician also suffers from migraine. In any case, it might be of interest to further investigate if the positive personal history of migraine among treating doctors may indeed improve the care or satisfaction of their patients with the same disease, as previously suggested in a French study surveying 711 general practitioners [19].

Previous evidence demonstrating that general practitioners may not manage migraine as well as they thought, resulting in increased rates of migraine misdiagnosis [20], further enhances the need for better training and a closer relationship between primary care physicians and headaches. Moreover, the results of a survey including 94 neurologists showed that migraine ranks among the most likeable diseases to be treated by neurology specialists [18]. Nonetheless, with our findings thoroughly demonstrating that most non-neurologist physicians, either general practitioners or internists working in primary care, remain reluctant to treat patients with migraine, we provide further evidence to support the claim that migraine currently remains both an underdiagnosed and undertreated headache disorder, as few patients are eventually referred from primary care physicians to neurologists, headache specialists, or headache clinics to be properly diagnosed and treated [21,22,23].

The difficulties in attending continued medical education seem to play a major role in explaining why primary care physicians remain reluctant to treat migraine. While, as previously mentioned, neurology is not formally included in the residency educational program of general practitioners and internists [9], Greek general practitioners were recently given the option to attend general neurology outpatient clinics in order to gain some experience in treating neurological disorders. Noteworthy, the interest of general practitioners in attending such optional educational programs remains low. Many of them preferred additional education in the management of other, more common diseases, thereby resulting in a low/moderate likeability to treat neurological diseases, including migraine.

Specifically concerning migraine, in a previous Greek survey among family care residents, 67% reported that they believed they had sufficient knowledge to diagnose a migraine attack, but only 27.5% felt comfortable treating migraine attacks. Not surprisingly, 30% of the participants in this survey reported that their neurological knowledge derives mostly from their undergraduate studies, 11.8% from personal study, 11.8% from their training in internal medicine, and the rest from a combination of all three, whereas a robust 86% of the participants agreed that they should receive formal training in neurology [24]. This situation concerning educational programs in neurology does not seem to be very different in other European countries or the USA. Although medical students routinely rotate through neurology, headache medicine is covered quite briefly, if at all. In the USA, general practitioners may have little training in neurology, limited to a 1-month, half-time rotation during residency [25]. Many non-neurologists, including primary care doctors, may be intimidated or uninterested in neurology during either medical school, residency, or in practice, and the term “neurophobia” refers to this commonly encountered situation [26,27,28]. Finally, Spanish family care practitioners also reported having difficulties investigating and treating patients with neurological disorders [29,30]. Taking all these data together, it is evident that the situation with education in neurological diseases among non-neurologist physicians in Greece is similar to that observed in other European countries and in the USA. Unfortunately, there is no current evidence for any further efforts to improve the education of Greek GPs or internists on neurological diseases, including the most common headache disorders.

As limitations of our survey, we acknowledge the following: (i) we have not assessed whether the low likeability of migraine or other neurological disorders among primary care physicians can affect patients’ satisfaction and management; (ii) a more in-depth subgroup analysis was lacking, as only gender and the presence of a personal headache history were evaluated.

## 5. Conclusions

Despite the fact that migraine is among the most common disorders encountered in primary care, family physicians in Greece report that they like to treat patients with migraine less than other general medicine conditions. Future studies are warranted to correlate the likeability of diseases by doctors with patient satisfaction and management, as this issue is of great interest to investigate. Moreover, further studies should be conducted in Greece as well as in other countries to determine if this dislike of treating migraines is related to a lack of education and knowledge on the topic or if other factors are also involved. Finally, it might be of great interest to look at the possible associations between treating preferences for a disease and skills, time spent with a patient, or empathy.

## Figures and Tables

**Table 1:** Demographic characteristics and work status of survey respondents.

Variable	Total (%)	Males (%)	Females (%)	*p*-Value
N	182 (100.0%)	110 (60.4%)	72 (39.6%)	
Age	49.6 ± 9.1	52.0 ± 9.8	46.1 ± 6.6	<0.001
Years of practice	13.8 ± 8.4	16.0 ± 10.4	10.6 ± 8.0	<0.001
Practice				
Private sector ^##^	91 (52.0%)	52 (30%)	39 (22.5%)	0.631
Public sector ^##^	82 (48.0%)	51 (30.1%)	31 (17.9%)	
Specialty				
General practitioners	95 (52.2%)	52 (28.6%)	43 (23.6%)	0.187
Internists working in primary care	87 (47.8%)	58 (31.8%)	29 (15.9%)	

^##^ Missing data from 9 participants who did not report if they work in the private or public sector.

**Table 2:** Prevalence of migraine and tension-type headache among survey respondents.

Variable	Total (%)	Males (%)	Females (%)	*p*-Value
N	182 (100.0%)	110 (60.4%)	72 (39.6%)	0.137
Migraine	15 (8.25%)	7 (3.85%)	8 (4.4%)	
Tension-type headache	19 (10.5%)	9 (5.0%)	10 (5.5%)	
No history	148 (81.3%)	94 (51.4%)	54 (29.8%)	

**Table 3:** Responses to diseases/symptoms, ranked by likeability.

Disease	Mean Response ± Standard Deviation			*p*-Value
	Total	Males	Females	
Hypertension	4.7 ± 0.6	3.6 ± 1.0	3.5 ± 1.1	0.695
Hyperlipidemia	4.6 ± 0.7	4.6 ± 0.6	4.7 ± 0.6	0.383
Upper respiratory tract infections	4.5 ± 0.7	4.5 ± 0.7	4.5 ± 0.7	0.533
Diabetes mellitus	4.4 ± 0.9	4.4 ± 0.9	4.3 ± 0.9	0.737
Low back pain	3.9 ± 0.9	3.9 ± 0.9	3.8 ± 1.1	0.429
Dizziness	3.9 ± 0.9	3.9 ± 0.9	3.9 ± 1.6	0.950
Transient ischemic attack	3.9 ± 1.0	3.9 ± 0.9	3.9 ± 1.2	0.960
Diabetic peripheral neuropathy	3.6 ± 1.0	3.6 ± 1.0	3.5 ± 0.6	0.574
Migraine	3.6 ± 1.0	3.6 ± 1.0	3.5 ± 1.1	0.695
Fibromyalgia	3.2 ± 1.1	3.4 ± 1.0	3.1 ± 1.1	0.147

## Data Availability

The corresponding author has full control of all primary data and agrees to allow the journal to review our data upon reasonable request.
